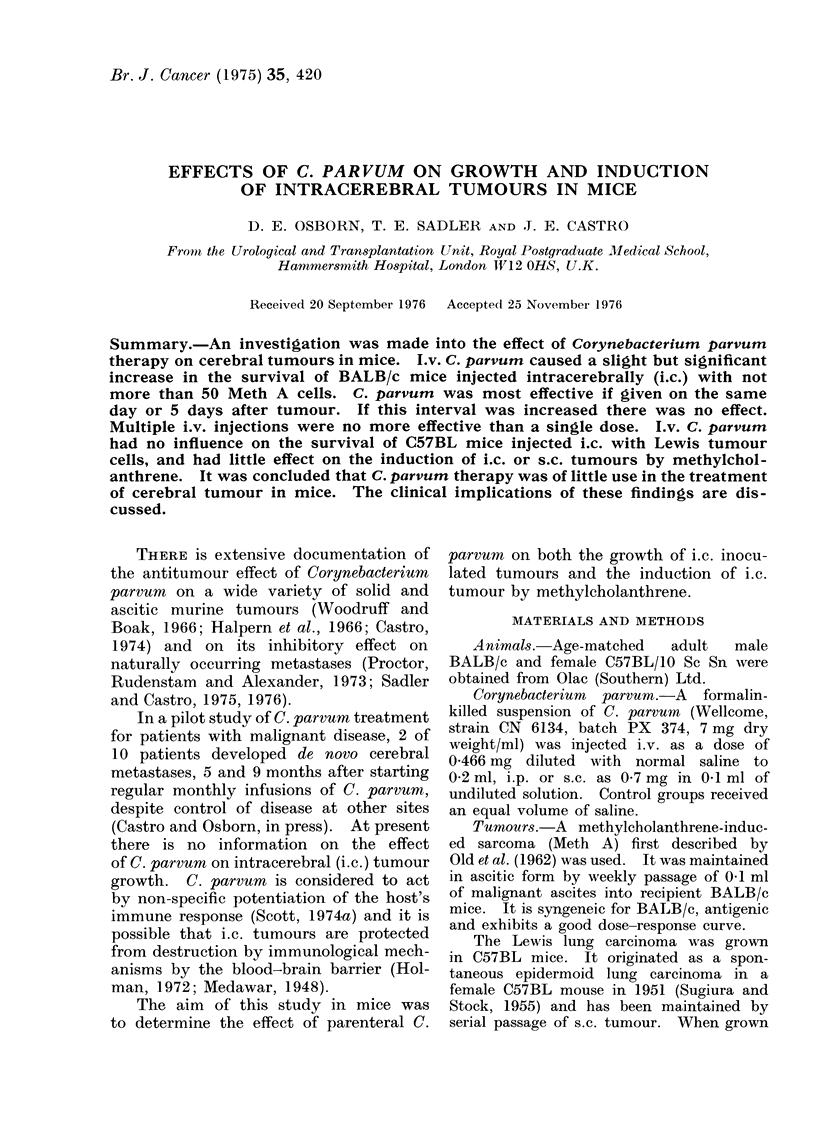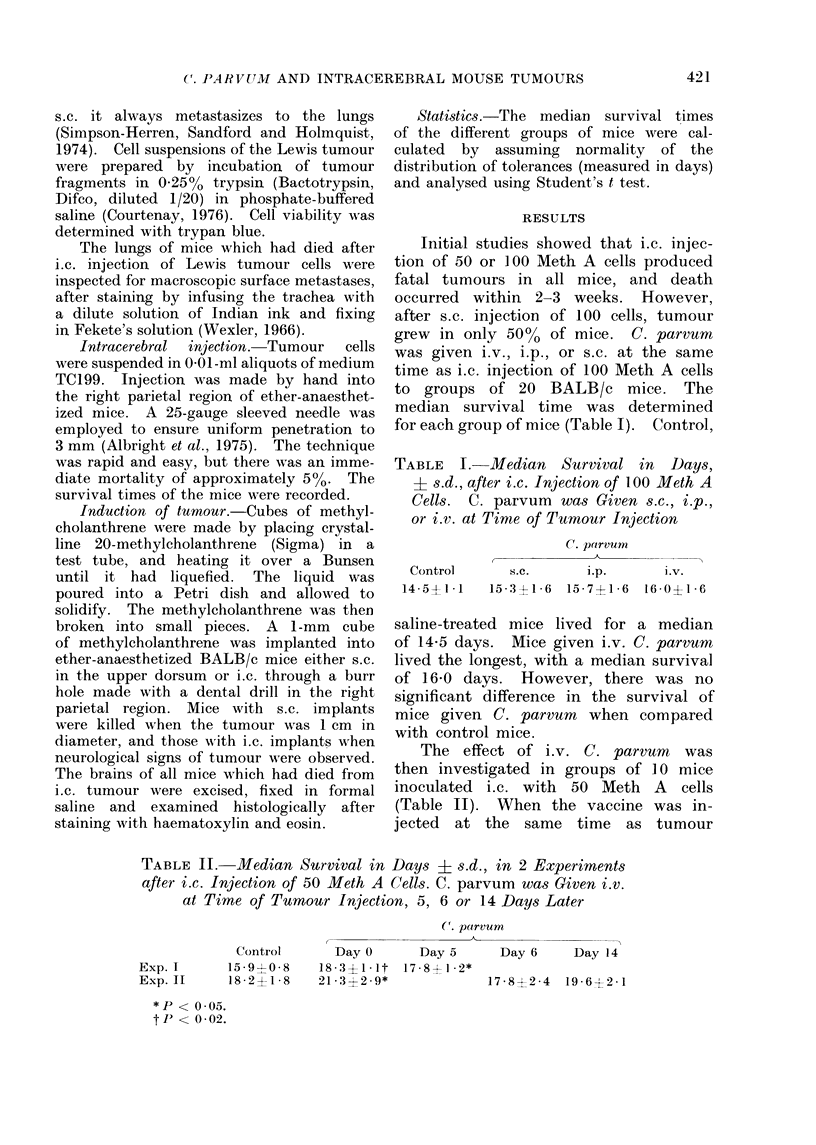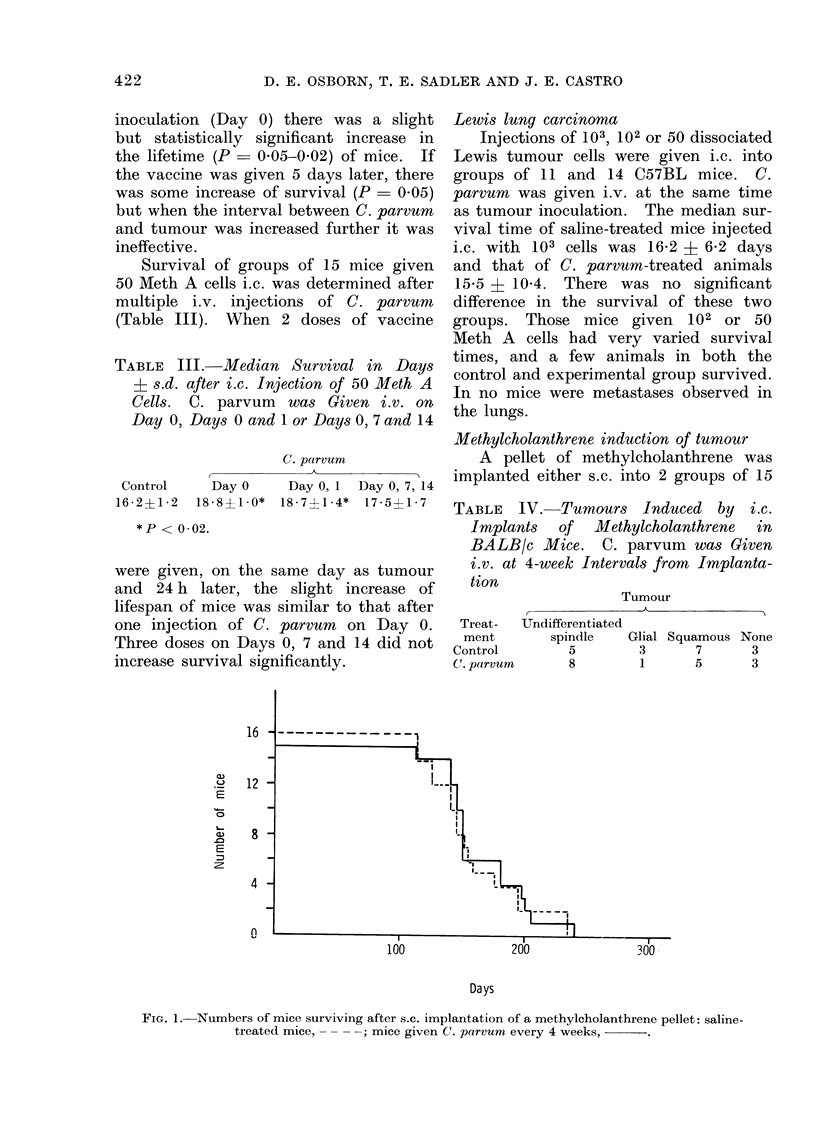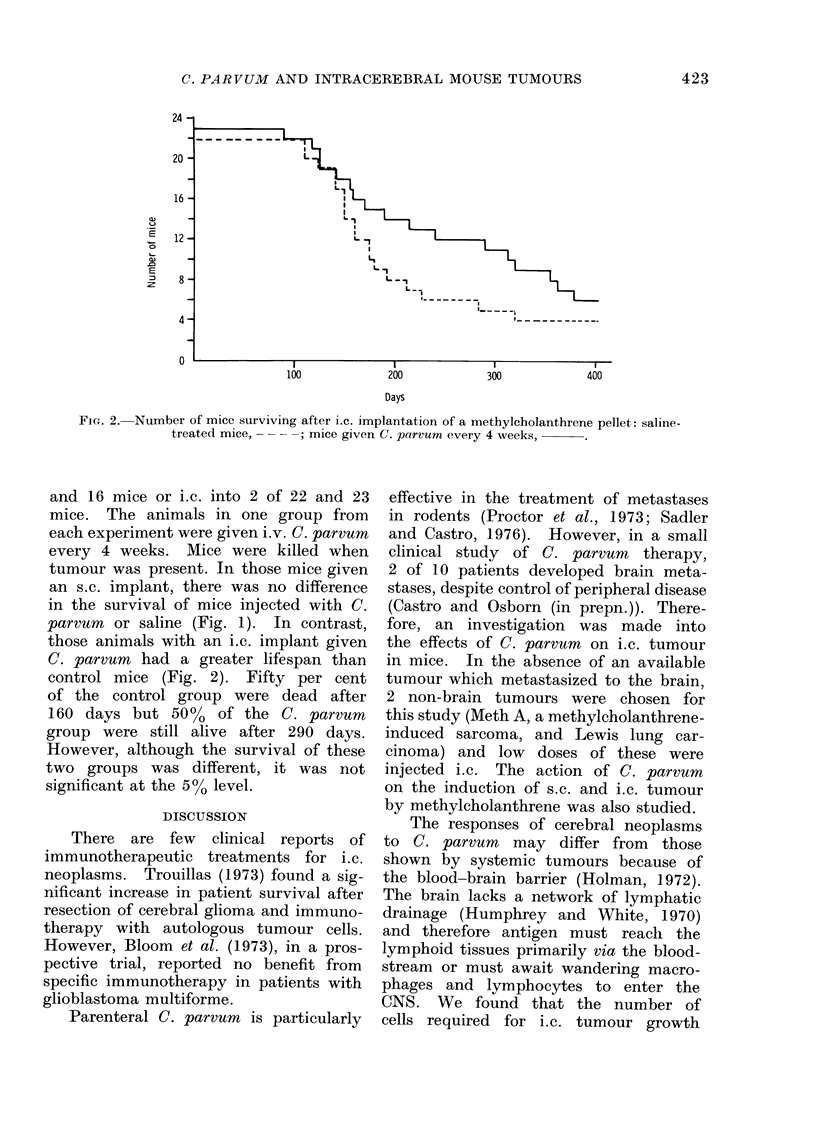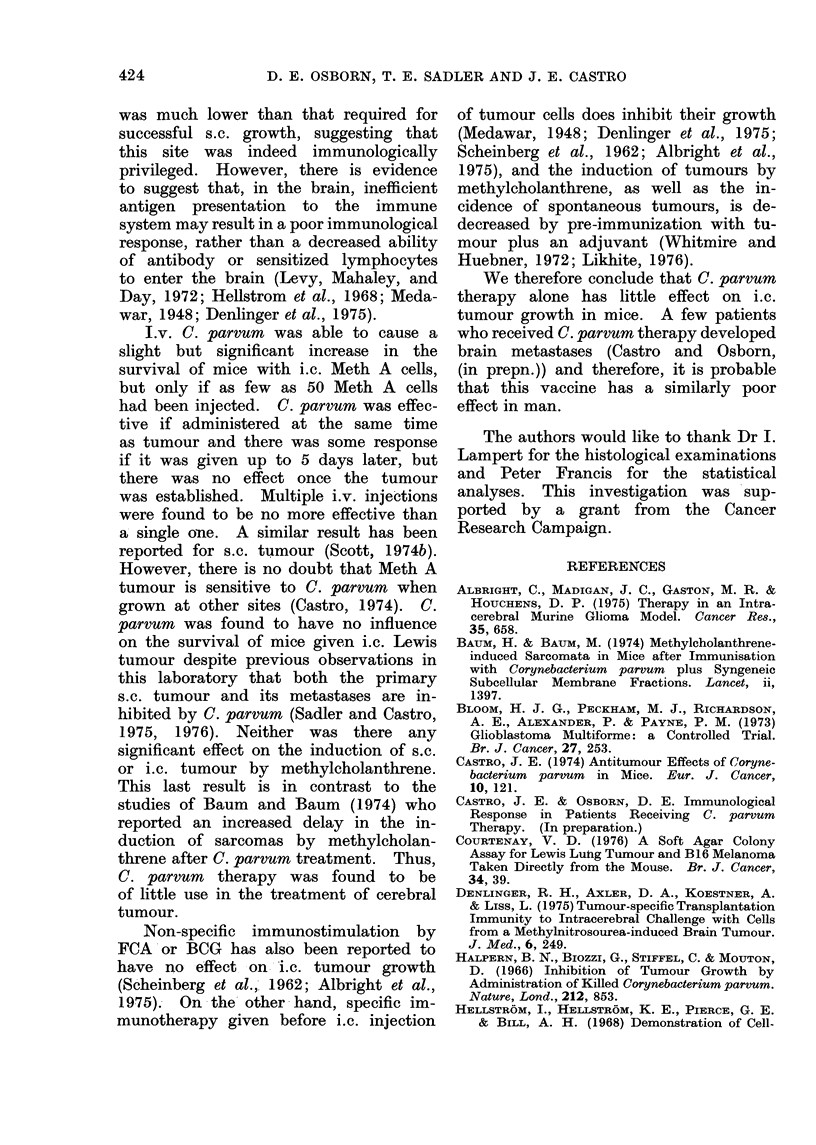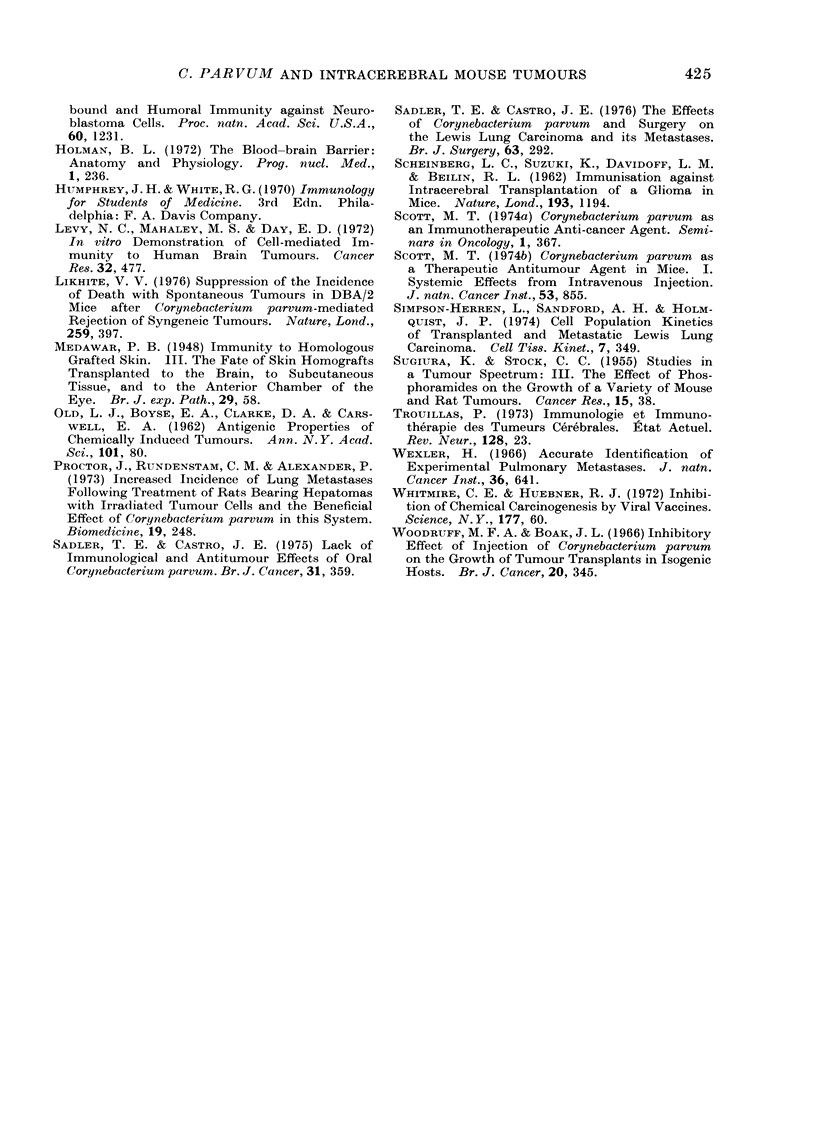# Effects of C. parvum on growth and induction of intracerebral tumours in mice.

**DOI:** 10.1038/bjc.1977.63

**Published:** 1977-04

**Authors:** D. E. Osborn, T. E. Sadler, J. E. Castro

## Abstract

An investigation was made into the effect of Corynebacterium parvum therapy on cerebral tumours in mice. I.v. C. parvum caused a slight but significant increase in the survival of BALB/c mice injected intracerebrally (i.c.) with not more than 50 Meth A cells. C. parvum was most effective if given on the same day or 5 days after tumour. If this interval was increased there was no effect. Multiple i.v. injections were no more effective than a single dose. I.v. C. parvum had no influence on the survival of C57BL mice injected i.c. with Lewis tumour cells, and had little effect on the induction of i.c. or s.c. tumours by methylcholanthrene. It was concluded that C. parvum therapy was of little use in the treatment of cerebral tumour in mice. The clinical implications of these findings are discussed.


*Br. J. Cancer* (1975) **35**, 420

### EFFECTS OF *C. PARVUM* ON GROWTH AND INDUCTION OF INTRACEREBRAL TUMOURS IN MICE

D. E. OSBORN, T. E. SADLER AND J. E. CASTRO

*From the Urological and Transplantation Unit, Royal Postgraduate Medical School, Hammersmith Hospital, London W12 0HS, U.K.*

Received 20 September 1976 Accepted 25 November 1976

**Summary.—An investigation was made into the effect of *Corynebacterium parvum* therapy on cerebral tumours in mice. I.v. *C. parvum* caused a slight but significant increase in the survival of BALB/c mice injected intracerebrally (i.c.) with not more than 50 Meth A cells. *C. parvum* was most effective if given on the same day or 5 days after tumour. If this interval was increased there was no effect. Multiple i.v. injections were no more effective than a single dose. I.v. *C. parvum* had no influence on the survival of C57BL mice injected i.c. with Lewis tumour cells, and had little effect on the induction of i.c. or s.c. tumours by methylcholanthrene. It was concluded that *C. parvum* therapy was of little use in the treatment of cerebral tumour in mice. The clinical implications of these findings are discussed.**

THERE is extensive documentation of the antitumour effect of *Corynebacterium parvum* on a wide variety of solid and ascitic murine tumours (Woodruff and Boak, 1966; Halpern *et al.*, 1966; Castro, 1974) and on its inhibitory effect on naturally occurring metastases (Proctor, Rudenstam and Alexander, 1973; Sadler and Castro, 1975, 1976).

In a pilot study of *C. parvum* treatment for patients with malignant disease, 2 of 10 patients developed *de novo* cerebral metastases, 5 and 9 months after starting regular monthly infusions of *C. parvum*, despite control of disease at other sites (Castro and Osborn, in press). At present there is no information on the effect of *C. parvum* on intracerebral (i.c.) tumour growth. *C. parvum* is considered to act by non-specific potentiation of the host's immune response (Scott, 1974*a*) and it is possible that i.c. tumours are protected from destruction by immunological mechanisms by the blood–brain barrier (Holman, 1972; Medawar, 1948).

The aim of this study in mice was to determine the effect of parenteral *C. parvum* on both the growth of i.c. inoculated tumours and the induction of i.c. tumour by methylcholanthrene.

### MATERIALS AND METHODS

*Animals.*—Age-matched adult male BALB/c and female C57BL/10 Sc Sn were obtained from Olac (Southern) Ltd.

*Corynebacterium parvum.*—A formalin-killed suspension of *C. parvum* (Wellcome, strain CN 6134, batch PX 374, 7 mg dry weight/ml) was injected i.v. as a dose of 0·466 mg diluted with normal saline to 0·2 ml, i.p. or s.c. as 0·7 mg in 0·1 ml of undiluted solution. Control groups received an equal volume of saline.

*Tumours.*—A methylcholanthrene-induced sarcoma (Meth A) first described by Old *et al.* (1962) was used. It was maintained in ascitic form by weekly passage of 0·1 ml of malignant ascites into recipient BALB/c mice. It is syngeneic for BALB/c, antigenic and exhibits a good dose–response curve.

The Lewis lung carcinoma was grown in C57BL mice. It originated as a spontaneous epidermoid lung carcinoma in a female C57BL mouse in 1951 (Sugiura and Stock, 1955) and has been maintained by serial passage of s.c. tumour. When grown s.c. it always metastasizes to the lungs (Simpson-Herren, Sandford and Holmquist, 1974). Cell suspensions of the Lewis tumour were prepared by incubation of tumour fragments in 0·25% trypsin (Bactotrypsin, Difco, diluted 1/20) in phosphate-buffered saline (Courtenay, 1976). Cell viability was determined with trypan blue.

The lungs of mice which had died after i.c. injection of Lewis tumour cells were inspected for macroscopic surface metastases, after staining by infusing the trachea with a dilute solution of Indian ink and fixing in Fekete's solution (Wexler, 1966).

*Intracerebral injection.*—Tumour cells were suspended in 0·01-ml aliquots of medium TC199. Injection was made by hand into the right parietal region of ether-anaesthetized mice. A 25-gauge sleeved needle was employed to ensure uniform penetration to 3 mm (Albright *et al.*, 1975). The technique was rapid and easy, but there was an immediate mortality of approximately 5%. The survival times of the mice were recorded.

*Induction of tumour.*—Cubes of methylcholanthrene were made by placing crystalline 20-methylcholanthrene (Sigma) in a test tube, and heating it over a Bunsen until it had liquefied. The liquid was poured into a Petri dish and allowed to solidify. The methylcholanthrene was then broken into small pieces. A 1-mm cube of methylcholanthrene was implanted into ether-anaesthetized BALB/c mice either s.c. in the upper dorsum or i.c. through a burr hole made with a dental drill in the right parietal region. Mice with s.c. implants were killed when the tumour was 1 cm in diameter, and those with i.c. implants when neurological signs of tumour were observed. The brains of all mice which had died from i.c. tumour were excised, fixed in formal saline and examined histologically after staining with haematoxylin and eosin.

*Statistics.*—The median survival times of the different groups of mice were calculated by assuming normality of the distribution of tolerances (measured in days) and analysed using Student's *t* test.

### RESULTS

Initial studies showed that i.c. injection of 50 or 100 Meth A cells produced fatal tumours in all mice, and death occurred within 2–3 weeks. However, after s.c. injection of 100 cells, tumour grew in only 50% of mice. *C. parvum* was given i.v., i.p., or s.c. at the same time as i.c. injection of 100 Meth A cells to groups of 20 BALB/c mice. The median survival time was determined for each group of mice (Table I). Control,

TABLE I.—*Median Survival in Days, ± s.d., after i.c. Injection of* 100 *Meth A Cells.* C. parvum *was Given s.c., i.p., or i.v. at Time of Tumour Injection*

| | *C. parvum* | | |
|---|---|---|---|
| Control | s.c. | i.p. | i.v. |
| 14·5±1·1 | 15·3±1·6 | 15·7±1·6 | 16·0±1·6 |

saline-treated mice lived for a median of 14·5 days. Mice given i.v. *C. parvum* lived the longest, with a median survival of 16·0 days. However, there was no significant difference in the survival of mice given *C. parvum* when compared with control mice.

The effect of i.v. *C. parvum* was then investigated in groups of 10 mice inoculated i.c. with 50 Meth A cells (Table II). When the vaccine was injected at the same time as tumour

TABLE II.—*Median Survival in Days ± s.d., in* 2 *Experiments after i.c. Injection of* 50 *Meth A Cells.* C. parvum *was Given i.v. at Time of Tumour Injection,* 5, 6 *or* 14 *Days Later*

| | | *C. parvum* | | | |
|---|---|---|---|---|---|
| | Control | Day 0 | Day 5 | Day 6 | Day 14 |
| Exp. I | 15·9±0·8 | 18·3±1·1† | 17·8±1·2* | | |
| Exp. II | 18·2±1·8 | 21·3±2·9* | | 17·8±2·4 | 19·6±2·1 |

inoculation (Day 0) there was a slight but statistically significant increase in the lifetime ($P = 0{\cdot}05$–$0{\cdot}02$) of mice. If the vaccine was given 5 days later, there was some increase of survival ($P = 0{\cdot}05$) but when the interval between *C. parvum* and tumour was increased further it was ineffective.

Survival of groups of 15 mice given 50 Meth A cells i.c. was determined after multiple i.v. injections of *C. parvum* (Table III). When 2 doses of vaccine were given, on the same day as tumour and 24 h later, the slight increase of lifespan of mice was similar to that after one injection of *C. parvum* on Day 0. Three doses on Days 0, 7 and 14 did not increase survival significantly.

TABLE III.—*Median Survival in Days ± s.d. after i.c. Injection of* 50 *Meth A Cells.* C. parvum *was Given i.v. on Day* 0, *Days* 0 *and* 1 *or Days* 0, 7 *and* 14

| | *C. parvum* | | |
|---|---|---|---|
| Control | Day 0 | Day 0, 1 | Day 0, 7, 14 |
| 16·2±1·2 | 18·8±1·0* | 18·7±1·4* | 17·5±1·7 |

* $P < 0{\cdot}02$.

*Lewis lung carcinoma*

Injections of $10^3$, $10^2$ or 50 dissociated Lewis tumour cells were given i.c. into groups of 11 and 14 C57BL mice. *C. parvum* was given i.v. at the same time as tumour inoculation. The median survival time of saline-treated mice injected i.c. with $10^3$ cells was $16{\cdot}2 \pm 6{\cdot}2$ days and that of *C. parvum*-treated animals $15{\cdot}5 \pm 10{\cdot}4$. There was no significant difference in the survival of these two groups. Those mice given $10^2$ or 50 Meth A cells had very varied survival times, and a few animals in both the control and experimental group survived. In no mice were metastases observed in the lungs.

*Methylcholanthrene induction of tumour*

A pellet of methylcholanthrene was implanted either s.c. into 2 groups of 15

TABLE IV.—*Tumours Induced by i.c. Implants of Methylcholanthrene in BALB/c Mice.* C. parvum *was Given i.v. at 4-week Intervals from Implantation*

| | Tumour | | | |
|---|---|---|---|---|
| Treatment | Undifferentiated spindle | Glial | Squamous | None |
| Control | 5 | 3 | 7 | 3 |
| *C. parvum* | 8 | 1 | 5 | 3 |

FIG. 1.—Numbers of mice surviving after s.c. implantation of a methylcholanthrene pellet: saline-treated mice, – – – –; mice given *C. parvum* every 4 weeks, ———.

FIG. 2.—Number of mice surviving after i.c. implantation of a methylcholanthrene pellet: saline-treated mice, - - - -; mice given *C. parvum* every 4 weeks, ———.

and 16 mice or i.c. into 2 of 22 and 23 mice. The animals in one group from each experiment were given i.v. *C. parvum* every 4 weeks. Mice were killed when tumour was present. In those mice given an s.c. implant, there was no difference in the survival of mice injected with *C. parvum* or saline (Fig. 1). In contrast, those animals with an i.c. implant given *C. parvum* had a greater lifespan than control mice (Fig. 2). Fifty per cent of the control group were dead after 160 days but 50% of the *C. parvum* group were still alive after 290 days. However, although the survival of these two groups was different, it was not significant at the 5% level.

### DISCUSSION

There are few clinical reports of immunotherapeutic treatments for i.c. neoplasms. Trouillas (1973) found a significant increase in patient survival after resection of cerebral glioma and immunotherapy with autologous tumour cells. However, Bloom *et al.* (1973), in a prospective trial, reported no benefit from specific immunotherapy in patients with glioblastoma multiforme.

Parenteral *C. parvum* is particularly effective in the treatment of metastases in rodents (Proctor *et al.*, 1973; Sadler and Castro, 1976). However, in a small clinical study of *C. parvum* therapy, 2 of 10 patients developed brain metastases, despite control of peripheral disease (Castro and Osborn (in prepn.)). Therefore, an investigation was made into the effects of *C. parvum* on i.c. tumour in mice. In the absence of an available tumour which metastasized to the brain, 2 non-brain tumours were chosen for this study (Meth A, a methylcholanthrene-induced sarcoma, and Lewis lung carcinoma) and low doses of these were injected i.c. The action of *C. parvum* on the induction of s.c. and i.c. tumour by methylcholanthrene was also studied.

The responses of cerebral neoplasms to *C. parvum* may differ from those shown by systemic tumours because of the blood–brain barrier (Holman, 1972). The brain lacks a network of lymphatic drainage (Humphrey and White, 1970) and therefore antigen must reach the lymphoid tissues primarily *via* the bloodstream or must await wandering macrophages and lymphocytes to enter the CNS. We found that the number of cells required for i.c. tumour growth was much lower than that required for successful s.c. growth, suggesting that this site was indeed immunologically privileged. However, there is evidence to suggest that, in the brain, inefficient antigen presentation to the immune system may result in a poor immunological response, rather than a decreased ability of antibody or sensitized lymphocytes to enter the brain (Levy, Mahaley, and Day, 1972; Hellstrom *et al.*, 1968; Medawar, 1948; Denlinger *et al.*, 1975).

I.v. *C. parvum* was able to cause a slight but significant increase in the survival of mice with i.c. Meth A cells, but only if as few as 50 Meth A cells had been injected. *C. parvum* was effective if administered at the same time as tumour and there was some response if it was given up to 5 days later, but there was no effect once the tumour was established. Multiple i.v. injections were found to be no more effective than a single one. A similar result has been reported for s.c. tumour (Scott, 1974*b*). However, there is no doubt that Meth A tumour is sensitive to *C. parvum* when grown at other sites (Castro, 1974). *C. parvum* was found to have no influence on the survival of mice given i.c. Lewis tumour despite previous observations in this laboratory that both the primary s.c. tumour and its metastases are inhibited by *C. parvum* (Sadler and Castro, 1975, 1976). Neither was there any significant effect on the induction of s.c. or i.c. tumour by methylcholanthrene. This last result is in contrast to the studies of Baum and Baum (1974) who reported an increased delay in the induction of sarcomas by methylcholanthrene after *C. parvum* treatment. Thus, *C. parvum* therapy was found to be of little use in the treatment of cerebral tumour.

Non-specific immunostimulation by FCA or BCG has also been reported to have no effect on i.c. tumour growth (Scheinberg *et al.*, 1962; Albright *et al.*, 1975). On the other hand, specific immunotherapy given before i.c. injection of tumour cells does inhibit their growth (Medawar, 1948; Denlinger *et al.*, 1975; Scheinberg *et al.*, 1962; Albright *et al.*, 1975), and the induction of tumours by methylcholanthrene, as well as the incidence of spontaneous tumours, is dedecreased by pre-immunization with tumour plus an adjuvant (Whitmire and Huebner, 1972; Likhite, 1976).

We therefore conclude that *C. parvum* therapy alone has little effect on i.c. tumour growth in mice. A few patients who received *C. parvum* therapy developed brain metastases (Castro and Osborn, (in prepn.)) and therefore, it is probable that this vaccine has a similarly poor effect in man.

The authors would like to thank Dr I. Lampert for the histological examinations and Peter Francis for the statistical analyses. This investigation was supported by a grant from the Cancer Research Campaign.

### REFERENCES

ALBRIGHT, C., MADIGAN, J. C., GASTON, M. R. & HOUCHENS, D. P. (1975) Therapy in an Intracerebral Murine Glioma Model. *Cancer Res.*, **35**, 658.

BAUM, H. & BAUM, M. (1974) Methylcholanthrene-induced Sarcomata in Mice after Immunisation with *Corynebacterium parvum* plus Syngeneic Subcellular Membrane Fractions. *Lancet*, ii, 1397.

BLOOM, H. J. G., PECKHAM, M. J., RICHARDSON, A. E., ALEXANDER, P. & PAYNE, P. M. (1973) Glioblastoma Multiforme: a Controlled Trial. *Br. J. Cancer*, **27**, 253.

CASTRO, J. E. (1974) Antitumour Effects of *Corynebacterium parvum* in Mice. *Eur. J. Cancer*, **10**, 121.

CASTRO, J. E. & OSBORN, D. E. Immunological Response in Patients Receiving *C. parvum* Therapy. (In preparation.)

COURTENAY, V. D. (1976) A Soft Agar Colony Assay for Lewis Lung Tumour and B16 Melanoma Taken Directly from the Mouse. *Br. J. Cancer*, **34**, 39.

DENLINGER, R. H., AXLER, D. A., KOESTNER, A. & LISS, L. (1975) Tumour-specific Transplantation Immunity to Intracerebral Challenge with Cells from a Methylnitrosourea-induced Brain Tumour. *J. Med.*, **6**, 249.

HALPERN, B. N., BIOZZI, G., STIFFEL, C. & MOUTON, D. (1966) Inhibition of Tumour Growth by Administration of Killed *Corynebacterium parvum*. *Nature, Lond.*, **212**, 853.

HELLSTRÖM, I., HELLSTRÖM, K. E., PIERCE, G. E. & BILL, A. H. (1968) Demonstration of Cellbound and Humoral Immunity against Neuroblastoma Cells. *Proc. natn. Acad. Sci. U.S.A.*, **60**, 1231.

Holman, B. L. (1972) The Blood–brain Barrier: Anatomy and Physiology. *Prog. nucl. Med.*, **1**, 236.

Humphrey, J. H. & White, R. G. (1970) *Immunology for Students of Medicine.* 3rd Edn. Philadelphia: F. A. Davis Company.

Levy, N. C., Mahaley, M. S. & Day, E. D. (1972) *In vitro* Demonstration of Cell-mediated Immunity to Human Brain Tumours. *Cancer Res.* **32**, 477.

Likhite, V. V. (1976) Suppression of the Incidence of Death with Spontaneous Tumours in DBA/2 Mice after *Corynebacterium parvum*-mediated Rejection of Syngeneic Tumours. *Nature, Lond.*, **259**, 397.

Medawar, P. B. (1948) Immunity to Homologous Grafted Skin. III. The Fate of Skin Homografts Transplanted to the Brain, to Subcutaneous Tissue, and to the Anterior Chamber of the Eye. *Br. J. exp. Path.*, **29**, 58.

Old, L. J., Boyse, E. A., Clarke, D. A. & Carswell, E. A. (1962) Antigenic Properties of Chemically Induced Tumours. *Ann. N.Y. Acad. Sci.*, **101**, 80.

Proctor, J., Rundenstam, C. M. & Alexander, P. (1973) Increased Incidence of Lung Metastases Following Treatment of Rats Bearing Hepatomas with Irradiated Tumour Cells and the Beneficial Effect of *Corynebacterium parvum* in this System. *Biomedicine*, **19**, 248.

Sadler, T. E. & Castro, J. E. (1975) Lack of Immunological and Antitumour Effects of Oral *Corynebacterium parvum*. *Br. J. Cancer*, **31**, 359.

Sadler, T. E. & Castro, J. E. (1976) The Effects of *Corynebacterium parvum* and Surgery on the Lewis Lung Carcinoma and its Metastases. *Br. J. Surgery*, **63**, 292.

Scheinberg, L. C., Suzuki, K., Davidoff, L. M. & Beilin, R. L. (1962) Immunisation against Intracerebral Transplantation of a Glioma in Mice. *Nature, Lond.*, **193**, 1194.

Scott, M. T. (1974*a*) *Corynebacterium parvum* as an Immunotherapeutic Anti-cancer Agent. *Seminars in Oncology*, **1**, 367.

Scott, M. T. (1974*b*) *Corynebacterium parvum* as a Therapeutic Antitumour Agent in Mice. I. Systemic Effects from Intravenous Injection. *J. natn. Cancer Inst.*, **53**, 855.

Simpson-Herren, L., Sandford, A. H. & Holmquist, J. P. (1974) Cell Population Kinetics of Transplanted and Metastatic Lewis Lung Carcinoma. *Cell Tiss. Kinet.*, **7**, 349.

Sugiura, K. & Stock, C. C. (1955) Studies in a Tumour Spectrum: III. The Effect of Phosphoramides on the Growth of a Variety of Mouse and Rat Tumours. *Cancer Res.*, **15**, 38.

Trouillas, P. (1973) Immunologie et Immunothérapie des Tumeurs Cérébrales. État Actuel. *Rev. Neur.*, **128**, 23.

Wexler, H. (1966) Accurate Identification of Experimental Pulmonary Metastases. *J. natn. Cancer Inst.*, **36**, 641.

Whitmire, C. E. & Huebner, R. J. (1972) Inhibition of Chemical Carcinogenesis by Viral Vaccines. *Science, N.Y.*, **177**, 60.

Woodruff, M. F. A. & Boak, J. L. (1966) Inhibitory Effect of Injection of *Corynebacterium parvum* on the Growth of Tumour Transplants in Isogenic Hosts. *Br. J. Cancer*, **20**, 345.